# The Molecular Mechanism of Yellow Mushroom (*Floccularia luteovirens*) Response to Strong Ultraviolet Radiation on the Qinghai-Tibet Plateau

**DOI:** 10.3389/fmicb.2022.918491

**Published:** 2022-06-20

**Authors:** Jing Guo, Zhanling Xie, Hongchen Jiang, Hongyan Xu, Baolong Liu, Qing Meng, Qingqing Peng, Yongpeng Tang, Yingzhu Duan

**Affiliations:** ^1^College of Ecological and Environment Engineering, Qinghai University, Xining, China; ^2^State Key Laboratory Breeding Base for Innovation and Utilization of Plateau Crop Germplasm, Qinghai University, Xining, China; ^3^State Key Laboratory of Biogeology and Environmental Geology, China University of Geosciences, Wuhan, China; ^4^Qinghai Provincial Key Laboratory of Geology and Environment of Salt Lakes, Qinghai Institute of Salt Lakes, Chinese Academy of Sciences, Xining, China; ^5^Academy of Agriculture and Forestry Sciences, Qinghai University, Xining, China; ^6^Key Laboratory of Adaptation and Evolution of Plateau Biota, Northwest Institute of Plateau Biology, Chinese Academy of Sciences, Xining, China; ^7^Bureau of Forestry and Grassland, Delingha, China; ^8^Test Station for Grassland Improvement, Xining, China

**Keywords:** *Floccularia luteovirens*, strong UV radiation, molecular regulation model, riboflavin, chlorophyll, Qinghai-Tibet Plateau

## Abstract

The Qinghai-Tibet Plateau (QTP) is the highest plateau in the world, and its ultraviolet (UV) radiation is much greater than that of other regions in the world. Yellow mushroom (*Floccularia luteovirens*) is a unique and widely distributed edible fungus on the QTP. However, the molecular mechanism of *F. luteovirens’s* response to strong UV radiation remains unclear. Herein, we reported the 205 environmental adaptation and information processing genes from genome of *F. luteovirens*. In addition, we assembled the RNA sequence of UV-affected *F. luteovirens* at different growth stages. The results showed that in response to strong UV radiation, a total of 11,871 significantly different genes were identified, of which 4,444 genes in the vegetative mycelium (VM) stage were significantly different from the young fruiting bodies (YFB) stage, and only 2,431 genes in the YFB stage were significantly different from fruiting bodies (FB) stage. A total of 225 differentially expressed genes (DEGs) were found to be involved in environmental signal transduction, biochemical reaction preparation and stress response pathway, pigment metabolism pathway, and growth cycle regulation, so as to sense UV radiation, promote repair damage, regulate intracellular homeostasis, and reduce oxidative damage of UV radiation. On the basis of these results, a molecular regulation model was proposed for the response of *F. luteovirens* to strong UV radiation. These results revealed the molecular mechanism of adaptation of *F. luteovirens* adapting to strong UV radiation, and provided novel insights into mechanisms of fungi adapting to extreme environmental conditions on the QTP; the production the riboflavin pigment of the endemic fungi (Yellow mushroom) in the QTP was one of the response to extreme environment of the strong UV radiation.

## Introduction

Ultravioet (UV) radiation is a component of solar radiation spectrum, which is the most harmful and mutagenic light band (Chen et al., 2021). UV can damage plant growth, development, biomass accumulation, and metabolism (Li et al., 2019a; Ozel et al., 2021). Plants have developed a series of strategies to cope with UV radiation, including activating light defense system, regulating light response pathway, and producing DNA photoproducts to mitigate DNA sequence changes and mutations caused by UV radiation (Azarafshan et al., 2020; Huarte-Bonnet et al., 2020). In addition, UV radiation will also activate the metabolic pathways of anthocyanin, carotenoid and melanin synthesis and anti-DNA oxidative damage system in plants, resulting in the accumulation of pigments such as anthocyanins, carotenoids, melanin, and riboflavin (Mapari et al., 2005; Gmoser et al., 2017; Qi et al., 2021; Zhu et al., 2021). In contrast for fungi, previous studies suggested that UV radiation would reduce the germination rate of conidia of *Magnaporthe oryzae*, and limited the population and diffusion of fungi in nature (Braga et al., 2015; Li et al., 2019a).

The Qinghai-Tibet Plateau (QTP) has the highest intensity of UV radiation in the world (Qiao et al., 2016). There are more than 1,500 kinds of fungi growing in the QTP alpine meadow ecosystem, among which yellow mushroom (*Floccularia luteovirens*) is one of the most flourish and representative macrofungi (Xing et al., 2017; Gan et al., 2020). In the alpine meadow of the QTP, our previous observation found that the wild fruiting bodies of *F. luteovirens* in high-UV environment were more yellowish brighter and showed more sulfur color than those in low-UV, and Liu et al. extracted riboflavin from yellow mushrooms fruiting bodies (Gan et al., 2022). However, the molecular adaptation mechanism of *F. luteovirens* in response to strong UV radiation remains unclear during its evolutionary process to extreme environment on QTP. In this work, we studied the gene expression of *F. luteovirens* at three growth stages [i.e., vegetative mycelium (VM) in lab, young fruiting body (YFB) in natural environment, fruiting body (FB) in natural environment] by using transcriptomics, and we also verified UV stress to cause the accumulation of the riboflavin and chlorophyll pigment in the vegetative mycelium, with the purpose to reveal the molecular adaptation mechanism of *F. luteovirens* to cope with strong UV radiation in the Qinghai-Tibet Plateau.

## Materials and Methods

### Vegetative Mycelium and Fruiting Body Material

In August 2018, three wild fruiting bodies of *F. luteovirens* were collected to transcriptome sequencing from Haiyan County (100°47′13″E, 37°0′37″N), Qinghai Province, China for transcriptome sequencing (Table 1). The sampling area is 3,220 m above sea level, with annual sun light of 2517.6–2995.3 h and annual average radiation of 5210.2–6568.3 MJ/m^2^ (Zhao Y. T. et al., 2021). According to the pileus diameter, the stage of *F. luteovirens* can be classified into young fruiting bodies (YFB, diameter 2.5–4.5 cm) and fruiting bodies (FB, diameter 4.5–6.6 cm), all of which samples were collected for transcriptome sequencing in three replicates. Vegetative mycelium (VM) was isolated from the fruiting bodies of *F. luteovirens* F18-3 and *F. luteovirens* DTS10, and were then cultivated in Potato Dextrose agar (PDA) medium (glucose 20.0 g/L, potato extract 200.0 g/L, agar 18.0 g/L) for 30 days at 20°C (at the same temperature of outside) in the dark (Xu et al., 2021). Three replicate plates were prepared for transcriptome sequencing and the determination of pigments. In the end of cultivation, the resulting *F. luteovirens* fruiting bodies were immediately frozen in liquid nitrogen and stored at −80°C.

**TABLE 1:** Sample information.

Strain number	Sample name	Sample size	Sample source	Environment	
F18-1	YFB	Pileus diameter is 2.5–4.5 cm			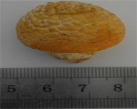
			Collected from HaiyanCounty from (100°47′ 13″E, 37°0′37″N, 3220 m), Qinghai Province, China, in August 2018	In nature: the annual sunlight radiation is 2517.6–2995.3 h; the average annual radiation is 5210.2 MJ– 6568.3 MJ/m2	
					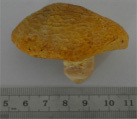
F18-2	FB	Pileus diameter is 4.5–6.6 cm		
F18-3	VM	Isolated from fruiting bodies of *F. luteovirens*		In lab: incubated in PDA medium at 20° for 30 days in the dark	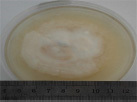

The strain of *F. luteovirens* F18-3 was cultured in PDB medium (glucose 20.0 g/L, potato extract 200.0 g/L) in flask of 500 mL at 200 rpm, at 20°C in liquid for 30 days. Three replicate plates in liquid culture were prepared for the determination of pigments.

### Genome Sequencing and Assembly

The genome of *F. luteovirens* fruiting body was sequenced with PacBio Sequel and Illumina Hiseq 2500 Systems (Genewiz Biotechnology Co. Ltd., Suzhou, China). Long reads from PacBio could give a high-quality sequencing length while also generating a 2.5-kb library. The genome size and hybrid rate of *F. luteovirens* was estimated by kmerfreq.^1^ Genome assembly was performed by using Canu software and was then assessed by using Benchmarking Universal SingleCopy Orthologs program v3.0 (Gan et al., 2020). The genome sequence of *F. luteovirens* has been deposited at GenBank under accession number of RPFY00000000.1.

### RNA Extraction and RNA-Sequencing

Total RNA was extracted from the three samples using TRIzol reagent, according to the manufacturer’s instructions (Sangon Biotech, Shanghai, China). The quality and quantity of total RNA were measured by using electrophoresis in 1% agarose gel. cDNA libraries were prepared using TruSeqTM RNA sample prep kit (Illumina). In brief, cDNA was synthesized from mRNA by reverse transcriptase with the use of random primers. Raw sequence reads were obtained by using an Illumina HiSeq sequencing platform. Each read was trimmed individually by setting quality score below 20 to obtain pure reads. Low quality reads were trimmed out and reads of 25 base pairs were filtered out in order to ensure purity. The sequences obtained in this study were deposited in the NCBI Sequence Read Archive (SRA^2^) under accession number SRP279887.

### *De novo* Transcriptome Assembly

Clean data were obtained by removing low-quality reads, adapter sequences, reads with ambiguous bases “N,” and reads of <20 bp. In the absence of a reference genome, contigs, and singletons were generated by de novo assembly using the Trinity software package.^3^

### Identification of Differentially Expressed Genes

Differentially expressed genes (DEGs) were identified by comparing gene expression levels using TPM (Transcripts Per Million). TPM is the most commonly used index of gene expression levels, and can reflect the impact of sequencing depth and gene length on read counts (Mortazavi et al., 2008). We used “*q* Value < 0.05 and | FoldChange| > 2” as the threshold to assess the significance of gene expression differences. BLAST analysis was carried out by searching against the NCBI databases and setting the E-value cut off at 10^–3^. The upregulated and downregulated unigenes were subject to Venn diagram analysis.

### Pathway Analysis of Differentially Expressed Genes

For metabolic pathway analysis, DEGs were aligned to the KEGG database through the KOBAS program.^4^ In both analyses, *q* value < 0.05 and | FoldChange| > 2 were used as thresholds.

### Quantitative Real-Time PCR Validation

Total RNA was extracted from same samples using a Fungal Total RNA Rapid Extraction Kit (Sangon Biotech, Shanghai, China). Then cDNA was synthesized by using an M-MuLV First Strand cDNA Synthesis kit (Sangon Biotech, Shanghai, China). The 18S ribosomal RNA (rRNA) gene was used as an internal control for normalization (Song et al., 2018). Primers were designed by primer 3.0 online^5^ (Supplementary Table 1). The qRT-PCR reaction was carried out in a LightCycler 96 system (Bio-Rad Laboratories, Hercules, CA, United States) using a SYBR Green kit (2 × SG Fast qPCR Master Mix, Sangon Biotech, Shanghai, China). Amplification conditions used for qPCR were 95°C for 3 min, followed by 40 cycles of 95°C for 3 s and 60°C for 30 s. For each sample, three biological and two technical replicates were done. Fold changes in gene expression were calculated using the 2^–ΔΔ*Ct*^ method (Li et al., 2019b).

### Determination of the Pigments on Riboflavin and Chlorophyll in Mycelia From Liquid and Solid Culture

The cultured mycelia of 30 days in liquid of F18-3 and solid culture of DTS10 were stressed by 5400 mW m^–2^ UV radiation for 0, 2, 4, 6, 8, 10, 12, 24 h. The mycelia from liquid culture and solid culture were collected and dry at 40°C for 2 h, and then at the weight of 100 mg mycelia were polished with liquid nitrogen for 5 min. For riboflavin measurements, samples were diluted to arrive at the linear range of the spectrophotometer with 0.05 M NaOH. The chlorophyll was determined according to the assay kits of chlorophyll (Nanjing Jiancheng Bioengineering Institute, Nanjing, China). The OD444 was immediately measured, the titer of riboflavin is calculated according to the following formula: y = 0.0105x + 0.0089, *R*^2^ = 0.9953 (Supplementary Figure 1; Zhang et al., 2021).

## Results

### Genome Assembly and Annotation

**FIGURE 1:**
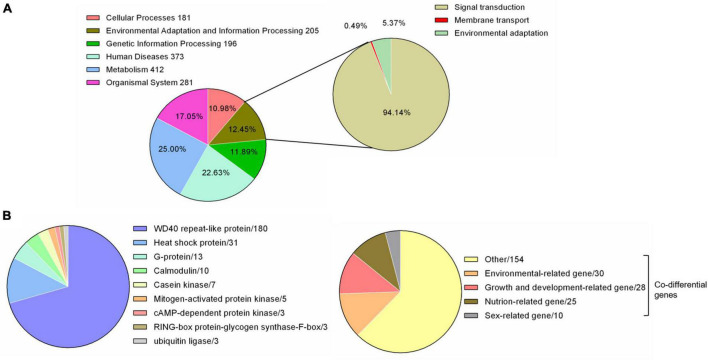
Environmental related genes of *F. luteovirens*. A total of 205–225 genes involved in responding to environment. **(A)** Pie chart of the 205 environmental adaptation and information processing genes from genome. **(B)** Pie chart of the 225 environmental related genes from transcriptome [The 225 genes = DEGs (255) − Co differential genes of environmental-related (30)].

The whole genome data of *F. luteovirens* were reported in previous studies (Gan et al., 2020). By comparing with 357 species-specific gene families from KEGG database, we found 1,648 genes in the whole genome of *F. luteovirens*. Among them, a total of 205 genes were annotated, which were categorized into three levels: 193 signal transduction genes (94.14%), one membrane transport gene (0.49%) and 11 environmental adaptation genes (5.37%), respectively (Figure 1A).

### Transcriptome Sequencing of *Floccularia luteovirens* and *de novo* Assembly

A total of 44,897,648, 53,152,546, and 46,470,082 high-quality sequence reads were obtained from the VM, YFB, and FB samples, respectively (Table 2). These reads were then assembled into 89962 extended transcripts with an average length of 2028.56 bp and a N50 length of 3559 bp (N50 indicates that at least half of the resulting transcripts have a base length of no less than 50 bp). The resulting extended transcripts were used to generate a transcriptome database by using Trinity (see text footnote 3). These extended transcripts consisted of 23823 unigenes, with an average unigene length of 1023.6 bp and a N50 length of 2447 bp (Table 3). The length of transcripts ranged from 200 to ≥2000 bp (Supplementary Figure 2).

**TABLE 2:** Throughput and quality of Illumina sequencing of the *Floccularia luteovirens* transcriptome.

Samples	Raw data			Quality control data		
	VM	YFB	FB	VM	YFB	FB
Total reads	46039192	54524788	47625192	44897648	53152546	46470082
Total length (bp)	6905878800	8178718200	7143778800	6385845795	7648146962	6699352210
Avg. length	150.0	150.0	150.0	142.23	143.89	144.16

**TABLE 3:** Functional annotation statistics of the *Floccularia luteovirens* assembly data.

Data	NO.	Total Len	Min Len	Max Len	Average	N50^c^
Transcript^a^	89962	182493102	201	18266	2028.56	3559
Unigene^b^	23823	24385122	201	18266	1023.6	2447

*^a^Total number of transcripts assembled using k-mer 51.*

*^b^Transcripts of multi-copy genes are collapsed into a single sequence.*

*^c^Half of all bases are in transcript at least as long as N50.*

### Analysis of Differentially Expressed Genes

**FIGURE 2:**
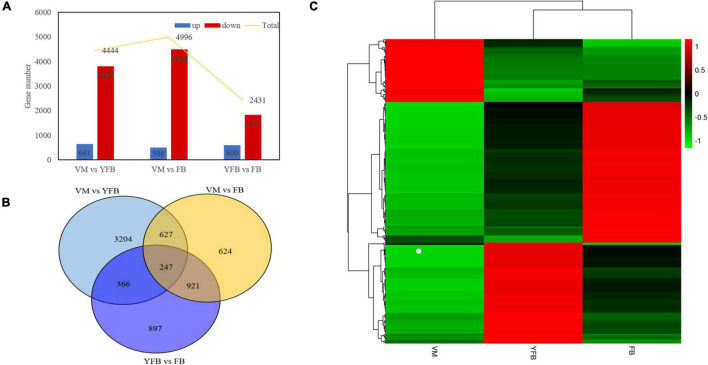
Bar plot, Venn diagram, and heatmap showing co-differentially expressed genes. **(A)** Bar plot of the differentially expressed genes (DEGs). **(B)** Venn diagram showing the DEGs exclusive to VM vs. YFB (light blue circle), YFB vs. FB (dark blue circle), and VM vs. FB (yellow circle). **(C)** Heatmap of co-differentially genes. Red and green colors in the heat-maps represent down- and up- regulated expression levels, respectively.

A total of 11,871 significant differently expressed genes (DEGs) were identified between the VM, YFB, and FB stages, of which 247 DEGs that were mainly involved in the growth and development of *F. luteovirens* co-existed in all three stages (Supplementary Table 2). A total of 4,444 DEGs were identified between the VM and YFB stages, 4,996 DEGs were identified between the VM and FB stages, and only 2,431 DEGs genes were identified between the YFB stage and FB stages (q ≤ 0.05) (Figure 2). Furthermore, cluster analysis showed the DEGs of YFB and FB belonged to the same branch, but the DEGs of VM formed a separate branch (Figure 2C), suggesting that the expression profiles of the YFB and FB were similar.

### Functional Analysis of Differentially Expressed Genes Under Strong Ultraviolet Radiation and External Environment Factors

**FIGURE 3:**
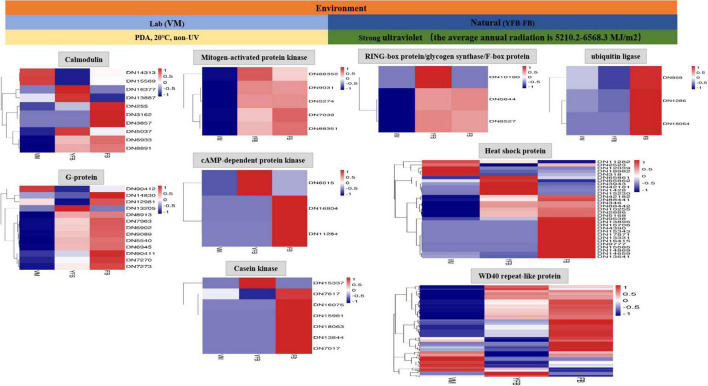
The 255 differential genes were annotated in response to UV radiation of *F. luteovirens*.

A total of 225 DEGs were identified to play a regulatory role in adapting to the changes of external environmental factors and UV radiation. The functions of these DEGs included signal transduction, biochemical reaction preparation and effect stress (Figure 1B and Supplementary Table 3). Among these identified DEGs, calmodulin and G proteins play important roles in signal transduction of *F. luteovirens*. Five calmodulin signaling genes that were up-regulated in the cells of *F. luteovirens* at the FB stage were identified, and they were calmodulin-binding motif protein (DN6933), calmodulin (DN255, DN3162) and Calcium/calmodulin-dependent protein (DN8891, DN3857). The expression of nine G protein coding genes were significantly up-regulated from the VM stage to the FB stage. Secondly, kinase genes are the key genes regulating the biochemical reaction in *F. luteovirens*. The expression of five mitogen-activated protein kinase genes were significantly up-regulated in the cells at the VM stage compared with those at the FB stage, the expression of two cAMP-dependent protein kinase genes, three casein kinase genes and cAMP-dependent protein kinase (DN6015) and casein kinase (DN15337) genes were significantly upregulated in the cells at the FB stage, compared with that in the VM stage. In addition, ubiquitin ligase, RING-box protein, glycogen synthase, and F-box protein were involved in the regulation of effector stress. In the present study, genes for ubiquitin ligases, glycogen synthase, and F-box protein were all expressed at a much higher level in the cells at the FB stage than that that of VM state. In addition, heat shock protein and WD40 repeat-like protein play vital role in oxidative stress of *F. luteovirens* under UV radiation and environmental factors. For instance, a large number of heat shock proteins were up-regulated in the cells at the FB growth stage. Most of the WD40 repeat-like protein genes of *F. luteovirens* were significantly upregulated in the cells at both YFB and FB stages (Figure 3).

### Metabolic Pathways Indicated by Differentially Expressed Genes

**FIGURE 4:**
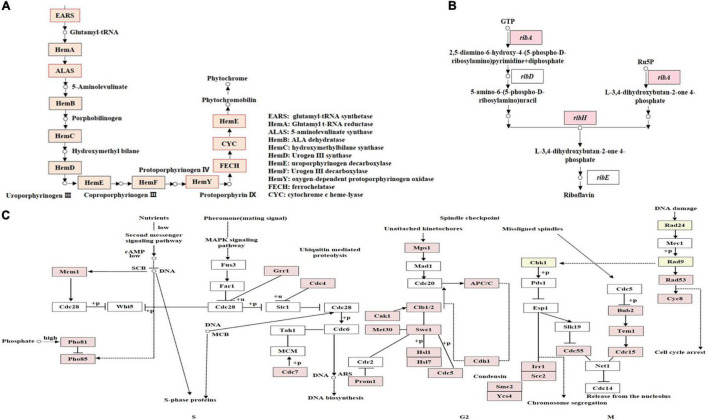
KEGG annotation of DEGs involved in different metabolism pathways. **(A)** KEGG annotation of proteins encoded by DEGs involved in the phytochrome biosynthesis pathway. The genes whose products are highlighted in red are up-regulated during the FB stages. **(B)** KEGG annotation of DEGs involved in riboflavin metabolism pathways. Genes highlighted in red are up-regulated during the FB stages. **(C)** KEGG annotation of DEGs involved in the cell-cycle. DEGs between VM and FB are shown in the cell-cycle pathway at the S, G2, and M phases. Genes whose products are highlighted in red are up-regulated during the FB stages.

Two metabolic pathways including chlorophyll and riboflavin metabolisms, related to pigment synthesis were differentially regulated in *F. luteovirens*. Several DEGs were enriched in the metabolisms of chlorophyll and riboflavin pathways. The chlorophyll metabolic pathway may affect the accumulation of photosynthetic pigments and the production of photochromes (Supplementary Figure 3). The gene expression of the core enzymes of the chlorophyll metabolic pathway was higher at the FB stage than at the VM stage (Supplementary Table 4). Such core enzyme included the genes of uroporphyrinogen decarboxylase (*HemE*, 21.24-fold), 5-aminolevulinate synthase (*ALAS*, 22.29-fold), ferrochelatase (*FECH*, 21.94-fold), glutamyl-tRNA synthetase (*EARS*, 21.41-fold), cytochrome c heme-lyase (*CYC*, 21.40-fold), oxygen-dependent protoporphyrinogen oxidase (*HemY*, 21.15-fold). In the riboflavin metabolism pathway, the expression levels of tyrosinase (*TYR*, 27.81-fold) and GTP cyclohydrolase II (*ribA*, 21.62-fold) genes were much higher than those in the FB stage than in the VM stage (Supplementary Table 5 and Supplementary Figure 4). Another gene for 3,4-dihydroxy 2-butanone 4-phosphate synthase (*ribB*) and 6,7-dimethyl-8-ribityllumazine synthase (*ribH*) was differentially expressed only in the FB stage (Figure 4B). The expression of these enzymes may result in the accumulation of riboflavin in *F. luteovirens* in the FB stage. In general, there were more differential regulation genes and specific genes of pigment synthesis under strong UV than without UV radiation, which was consistent with the fact that the fruiting body of *F. luteovirens* obtained long-term sunlight radiation and a large amount of UV radiation in the late stage of development.

In addition, the cell-cycle pathway of *F. luteovirens* was associated with its cell growth, proliferation, and development. The expression of the genes related to cell-cycle pathway in the cells of *F. luteovirens* was greatly up-regulated, and such genes included the S phase-related *Mcm1* (21.01-fold), *Pho81* (22.31-fold), *Pho85* (21.58-fold), *Grr1* (21.73-fold), *Cdc4* (21.42-fold), and *Cdc7* (23.88-fold); the G2 phase-related *Mps1* (21.70-fold), *Cak1* (22.02-fold), and *Cdh1* (21.67-fold) (Supplementary Figure 5); and the M phase-related *Rad53* (21.61-fold) and *Scc2* (21.46-fold), were higher in the FB stage than in the VM stage (Supplementary Table 6). However, the expression of *Cdc7* and *Cyc8* were significantly down-regulated, which were considered to depress the growth and development of *F. luteovirens* cells. Moreover, there were 34 DEGs that may be mainly involved in DNA damage repair of *F. luteovirens*, and they showed higher expression at the FB stage than at the VM stage (Figure 4C).

### Validation of RNA-Seq Results by qRT-PCR

**FIGURE 5:**
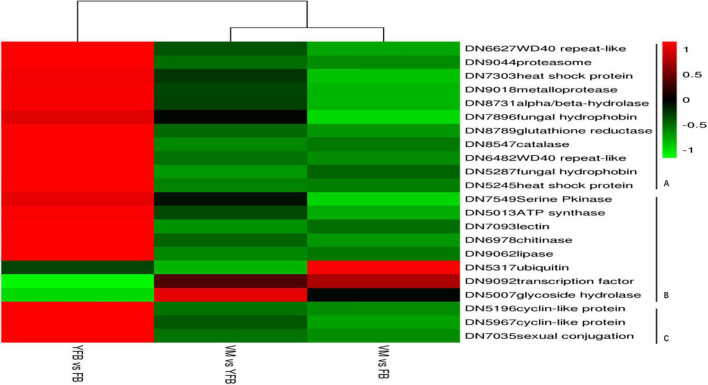
qRT-PCR validation of DEGs. The 18S gene was selected as the internal control in this study. The expression patterns determined by qRT-PCR were consistent with those obtained by RNA-Seq, thus confirming the reliability of our RNA-Seq data. **(A)** Eleven environment-related DEGs were analyzed by qRT-PCR. **(B)** Eight growth and development-related DEGs were analyzed by qRT-PCR. **(C)** Three sex-related DEGs were analyzed by qRT-PCR.

Twenty-two DEGs from *F. luteovirens* that were significantly expressed at all the three stages (VM, YFB, and FB) were randomly selected, and their differential expression was confirmed by qRT-PCR (Supplementary Table 7). Among them, 11 environment-related DEGs, eight growth and development-related DEGs, and three sex-related DEGs were investigation, all of which have different expression patterns similar to those in the RNA-Seq (Figure 5), indicating the reliability and accurately of the RNA-Seq data of *F. luteovirens*.

### Effect of Ultraviolet on Riboflavin and Chlorophyll Production in *Floccularia luteovirens* Mycelium

**FIGURE 6:**
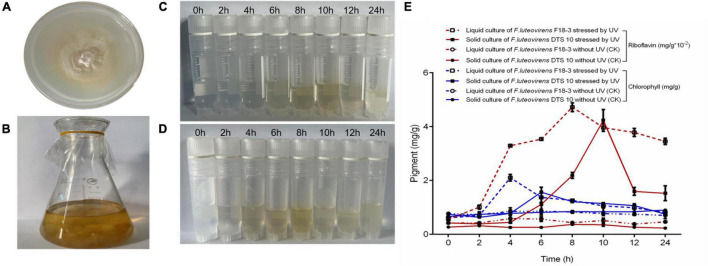
Riboflavin and chlorophyll induced by UV of *F. luteovirens* in mycelium. **(A)** Plate culture of *F. luteovirens* F18-3. **(B)** Liquid culture of *F. luteovirens* DTS10. **(C)** Abstracted riboflavin of *F. luteovirens* F18-3 in mycelium stressed by UV in solid culture. **(D)** Abstracted riboflavin of *F. luteovirens* DTS10 in mycelium stressed by UV in liquid culture. **(E)** The production of riboflavin and chlorophyll stressed by UV of *F. luteovirens* in mycelium. The red line represents riboflavin; the blue line represents chlorophyll.

Under UV irradiation for 8–10 h, the riboflavin concentration of mycelium reached the concentration of 42.35–47.21 ug/mL (Figure 6). Meanwhile, the chlorophyll content reached the 1.56–2.10 mg/g after UV stress for 4–6 h. Both DTS 10 and F18-3 of *F. luteovirens* reached the highest concentration after simultaneous exposure to UV radiation of 5400 mW m^–2^, although they were cultured and exposed in different states. The highest of riboflavin concentration occurred at UV radiation of 8–10 h, which was consistent with the daytime illumination time of *F. luteovirens*.

## Discussion

UV radiation affected the biochemical process and physiological characteristics of organisms. In this study, 205–225 DEGs potentially involved in coping with external strong UV radiation stress were obtained from *F. luteovirens* living on the Qinghai-Tibet Plateau. The genome environmental adaptation and information processing genes of these DEGs were basically consistent with the transcriptome genes of their environment-related genes. When *F. luteovirens* cells received signals of UV and external stimuli, G protein and calmodulin were activated as signal molecules. G protein interacted directly with downstream enzymes or ion channels and was involved in regulating cell-specific changes caused by UV and external stimuli (Smrcka and Fisher, 2019; Wang et al., 2019; Patel et al., 2020). Calmodulin, as a ubiquitous sensor protein and calcium receptor in eukaryotic cells, was involved in regulating the response of organisms to various environmental stresses and transmitting stimulation signals (Kiselev et al., 2021; Li et al., 2021). Our results showed that the significantly upregulated genes of DN255 and DN90411 were considered as candidate genes that played a crucial role in coping with UV stress and signal transduction in response to external factors. This finding was consistent with the expression trend of signal transduction genes of *CaM* and *CML* in *Vitis amurensis* under abiotic stress (Kiselev et al., 2021).

In addition, phosphorylation pathway is a key biological process in signal transduction, and can regulate the expression of kinase genes. When the kinase genes sense stimulation signals, cAMP-dependent protein kinase was the core enzyme that regulated the protein phosphorylation pathway (Wu Z. et al., 2019), and MAPK transduced signals through the phosphorylation pathway of downstream targets (Li C. et al., 2018). DEGs of DN7039 and DN88351 were found to be involved in the regulation of UV and external stress response of *F. luteovirens* and showed differential expression patterns. Furthermore, casein kinase is another gene essential for cell proliferation. It is an evolutionarily conserved Ser/Thr protein kinase and is also pivotal in signal transduction (Li Y. et al., 2018). Nagatoshi et al. (2018) reported that casein kinase is a pleiotropic enzyme involved in a variety of developmental and stress response processes. Our results suggested that casein kinase may be independently involved in signal transduction of *F. luteovirens* on the Qinghai-Tibet Plateau.

The cells of *F. luteovirens* often undergo post-translational modifications of proteins they sense and transmit internal or external UV stimulation signals (Han et al., 2019; Jiao and Duan, 2019; Yu et al., 2021). In *F. luteovirens*, RING-box protein, ubiquitin ligase, F-box protein, and glycogen synthase were involved in the post-translational modification of proteins under UV and external stimulation: on the one hand, RING-box proteins and ubiquitin ligases were identified as key regulators of basic cellular processes and participated in plant growth and development (Abd-Hamid et al., 2020); On the other hand, F-box protein and glycogen synthase depended on the regulation of protein stability under abiotic stress, and participated in the regulation of antioxidant system after translational modification (Rameneni et al., 2018; Jiao and Duan, 2019). Thus it is reasonable to speculate that RING-box protein and ubiquitin ligase may mediate the ubiquitination of key factors in the process of UV and external stress, and then regulate the adaptability of *F. luteovirens* to strong UV radiation and external factors.

Heat shock protein and WD40-repeat like protein were important molecular regulators involved in controlling cellular processes in response to stresses (Guz et al., 2021; Kim et al., 2021). The former helped to maintain the folding of damaged and newly formed proteins under stress conditions, and also responded to environmental stress by regulating the expression dynamics of numerous genes involved in maintaining intracellular homeostasis (Guz et al., 2021; Kim et al., 2021); while the latter mainly regulated signal transduction, transcriptional regulation, and immune response (Jain and Pandey, 2018). Our results suggested that under UV the stimulation and external factors, *F. luteovirens* cells produced denatured peptides and new proteins, so the genes of DN13641 and DN7704 were significantly upregulated.

Chlorophyll metabolism includes chlorophyll biosynthesis, chlorophyll cycle, and chlorophyll degradation. The produced chlorophyll can regulate the host’s defense response and survival under different extreme environmental conditions (Wang et al., 2020). In this study, 20 candidate genes related to porphyrin and chlorophyll metabolic pathway were analyzed. It is found that the core genes of *HemE*, *ALAS*, *FECH*, *EARS*, *CYC*, *HemY* were involved in the synthesis of phytochrome and cytochrome in *F. luteovirens* under strong UV radiation. Previous studies showed that *ALAS* can promote plant growth under abiotic stress and participate in the regulation of plant photosynthesis, nutrient absorption, antioxidant properties and osmotic balance (Wu Y. et al., 2019). Therefore, *ALAS* may mediate the color change of *F. luteovirens* to adapt to the strong UV radiation on the Qinghai-Tibet Plateau. The role of *ALAS* in *F. luteovirens* may be consistent with that of HY5 in regulating the color change of *Brassica campestris L.* leaf under cold stress (Yuan et al., 2021).

In addition to chlorophyll, riboflavin also participated in the response to UV radiation and the other external environment stimuli in the Qinghai-Tibet Plateau. The coding genes of FXN and BLVRA, the main derivatives of riboflavin, were other genes that mainly regulate plant growth and color rendering (Bai et al., 2019; Rouault, 2019; Britti et al., 2021). We found that the coding genes of FXN and BLVRA were involved in controlling the growth and division of *F. luteovirens* under strong UV radiation, and regulated its yellow color at the later growth stage of *F. luteovirens* (Figure 4A). FMN and FAD, the main riboflavin derivatives, were cofactors of enzymes that mediated many redox reactions in cells, and riboflavin operon *ribA* gene encoded GTP, which was the first step of riboflavin biosynthesis (Cisternas et al., 2017; Liu et al., 2020; Zhao G. et al., 2021). Accordingly, we found that *F. luteovirens* may have the capability of *de novo* riboflavin biosynthesis, and the *ribA* and *ribH* genes were directly involved in riboflavin synthesis. Their differential expression patterns may be related to the regulation of redox reaction and electron respiratory chain transmission under UV radiation. This is similar to the role of the anthocyanins of *Lycium ruthenicum* on Qinghai-Tibet Plateau (Qi et al., 2021). These results suggested that the metabolic pathway of fungal pigment synthesis was closely related to the response to UV radiation.

Furthermore, fungi have evolved complex signal transduction pathways to repair direct DNA damage. DNA damage checkpoints transmit signals of damaged DNA to effector molecules, thereby regulating cell cycle pathways to repair DNA oxidative damages (Zhou et al., 2016; Gkouskou et al., 2020). In the cell cycle pathway of *F. luteovirens*, the key genes *Rad53*, *Pho85*, *Pho81*, and *Cdh1* were significantly upregulated in the FB stage under strong UV radiation. The *Rad53* gene was responsible for the transduction of UV stimulated signals and was significantly upregulated under strong UV radiation to regulate DNA damage repair, replication, bifurcation stability, cell cycle progression, and transcription (Gkouskou et al., 2020). *Pho81* and *Pho85* gene were significantly upregulated in *F. luteovirens*, indicating that their encoded enzymes may play a key role in maintaining phosphate homeostasis under UV stimulation; *Pho85* controlled the cell cycle by regulating phosphate metabolism when it binds to the *Pho80* family of cyclins, while the CDK inhibitor *Pho81* regulated the cell cycle by inhibiting thePho80-Pho85 complex (Zhou et al., 2016; Zheng et al., 2020). The *Cdh1* gene encoded cell division cycle 20-like protein 1, which was highly upregulated in the FB stage under strong UV radiation. This result suggested that *Cdh1* may be a key regulatory gene in the cell cycle of the fruiting body of *F. luteovirens* from the S phase to G2 phase, which may be consistent with the role of *Chl1p* as G1/S phase checkpoint and its role in the damage checkpoint pathway in budding yeast (Katheeja et al., 2021).

## Conclusion

**FIGURE 7:**
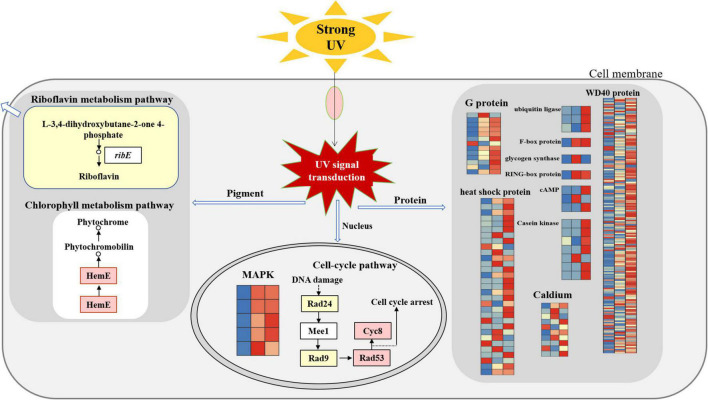
The molecular regulation mode of *F. luteovirens* under UV radiation.

*Floccularia luteovirens* can adapt to the changes of external environmental factors, especially it can respond to UV radiation in three ways by regulating key genes and obtain the ability to deal with UV radiation (as shown in the conceptual scheme Figure 7). Firstly, at the nuclear level, cell cycle was regulated to repair DNA oxidative damage of ultraviolet radiation to cells. Secondly, at the cytoplasmic level, differentially expressed genes of environmental signal transduction, biochemical reaction preparation and stress response involved in maintaining intracellular homeostasis, and the metabolic pathways of chlorophyll and riboflavin were activated to involved in the pigment synthesis of ultraviolet radiation to cells. In addition, after metabolizing gene clusters at the fruiting body and mycelium levels, the pigment of yellowish brightness and sulfur color emerged in the cells of yellow mushroom, which contribute to the characteristic yellow color of ultraviolet radiation to cells. This regulation model can reveal the molecular mechanism of *F. luteovirens* to deal with the strong UV radiation, which is of great significance for us to understand the environmental adaptation mechanism of fungi on the Qinghai-Tibet Plateau.

## Data Availability Statement

The datasets presented in this study can be found in online repositories. The names of the repository/repositories and accession number(s) can be found below: NCBI–RPFY00000000.1, SRP279887.

## Author Contributions

JG: conceptualization, methodology, and writing-original draft. ZX: supervision, validation, and writing-review and editing. HJ: writing-review and editing. HX: investigation. BL: supervision. QM: visualization. All authors contributed to the article and approved the submitted version.

## Conflict of Interest

The authors declare that the research was conducted in the absence of any commercial or financial relationships that could be construed as a potential conflict of interest.

## Publisher’s Note

All claims expressed in this article are solely those of the authors and do not necessarily represent those of their affiliated organizations, or those of the publisher, the editors and the reviewers. Any product that may be evaluated in this article, or claim that may be made by its manufacturer, is not guaranteed or endorsed by the publisher.

## Footnotes

1
https://github.com/fanagislab/kmerfreq

2
http://www.ncbi.nlm.nih.gov/Traces/sra

3
https://github.com/trinityrnaseq/trinityrnaseq/wiki

4
http://kobas.cbi.pku.edu.cn/kobas3’

5
https://primer3.ut.ee/
