# Supplementary material for: The Molecular Mechanism of Yellow Mushroom (Floccularia luteovirens) Response to Strong Ultraviolet Radiation on the Qinghai-Tibet Plateau
Source: Front Microbiol. 2022 Jun 20;13:918491. doi: 10.3389/fmicb.2022.918491 (PMC9251379; doi:10.3389/fmicb.2022.918491)
Supplement: Supplementary file 1 [file Table_1.DOCX]

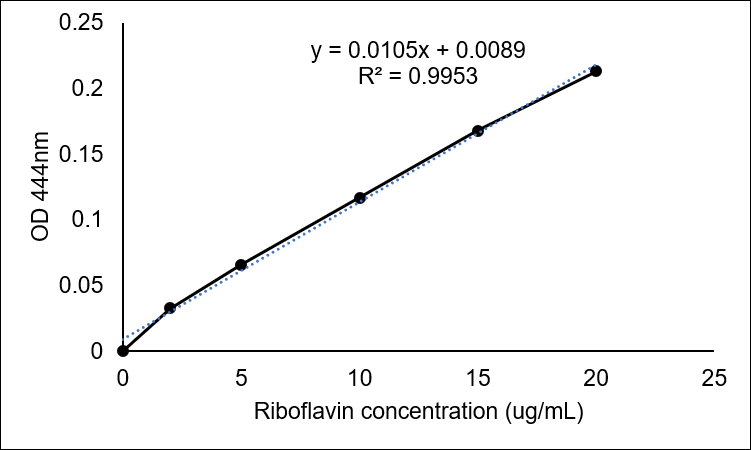


**Fig.S1 Standard curve of riboflavin**

**Fig.S2 Length distribution of transcripts assembled from the transcriptomes of the *F.luteovirens*.** The horizontal axis shows the lengths (base pairs) of transcripts and representative transcripts, and the vertical axis shows the number of total transcripts and representative transcripts.


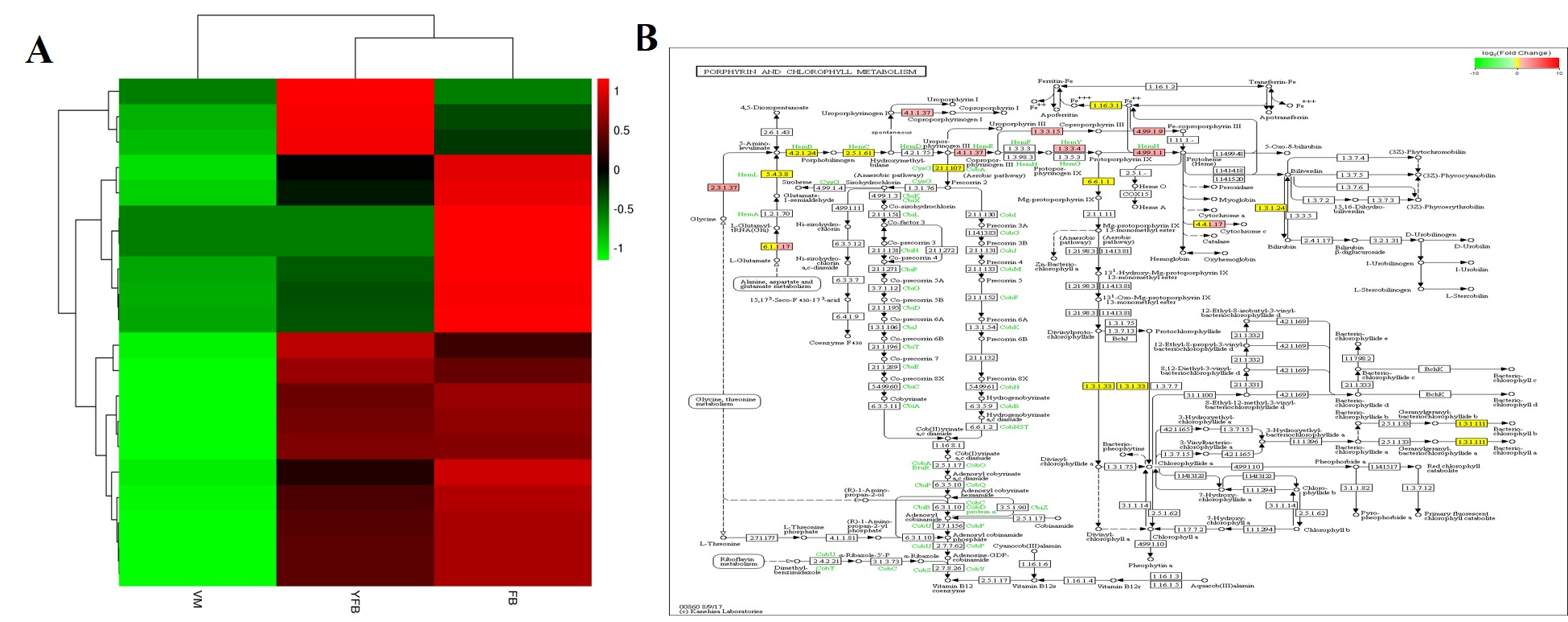


**Fig.S3 Heatmap analysis of the unigenes involved in** [**chloroohyll metabolism**](https://www.sciencedirect.com/topics/biochemistry-genetics-and-molecular-biology/purine-metabolism) **and KEGG annotation of the DEGs involved in the porphyrin and chlorophyll metabolism pathway.** (A)The heatmap shows the unigenes involved in chlorophyll metabolism at the three developmental stages on the basis of KEGG database analysis. The color bar reflect gene expression leveles, from green (low) to red (high). (B)DEGs between VM and FB stages in the porphyrin metabolism pathway are annotated. Boxes with red frames represent up-regulated genes; boxes with yellow frames represent non DEGs.


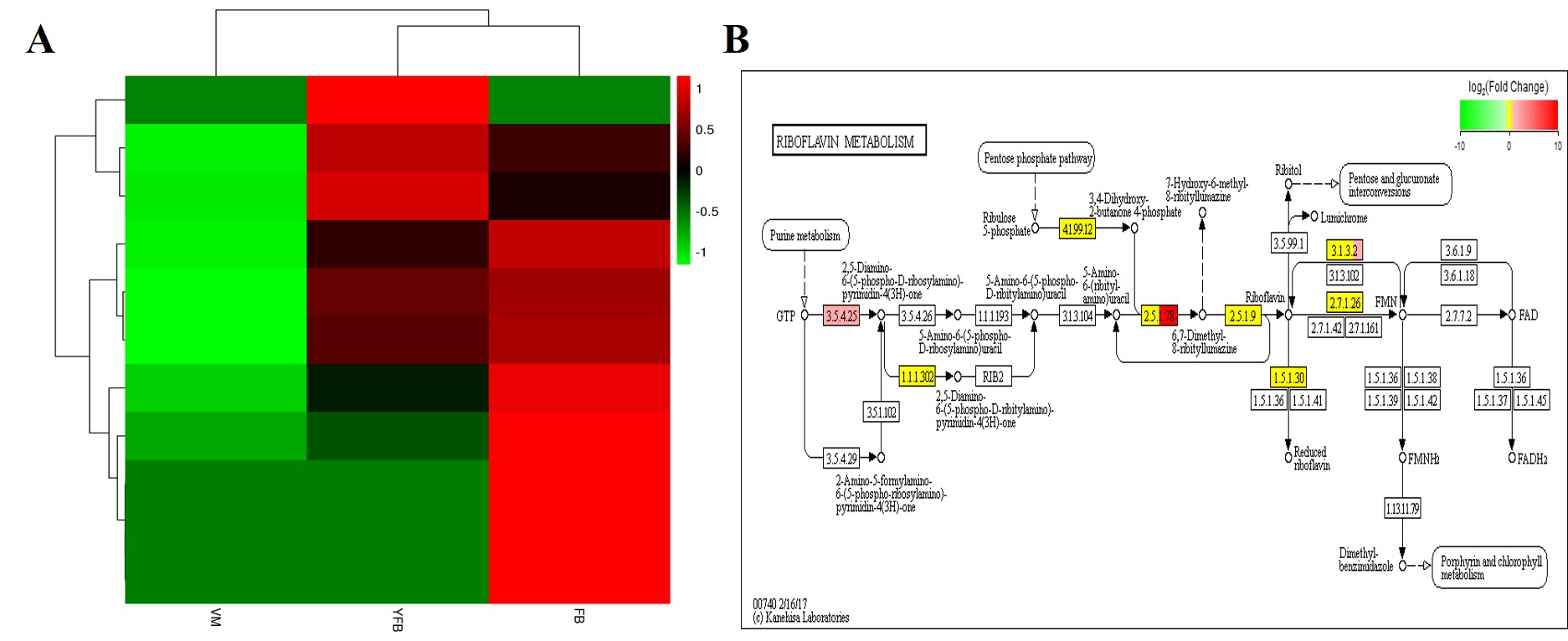


**Fig.S4 Heatmap analysis of unigenes involved in riboflavin metabolism and KEGG annotation of DEGs involved in the riboflavin metabolism pathway.** (A)The heatmap shows the unigenes involved in riboflavin metabolism according to the KEGG database at the three stages. The color bar reflects gene expression levels, from green(low) to red (high). (B) DEGs between the VM and FB stages in the riboflavin metabolism pathway are annotated. Boxes with red frames represent up-regulated genes; boxes with yellow frames represent genes showing no difference in expression.


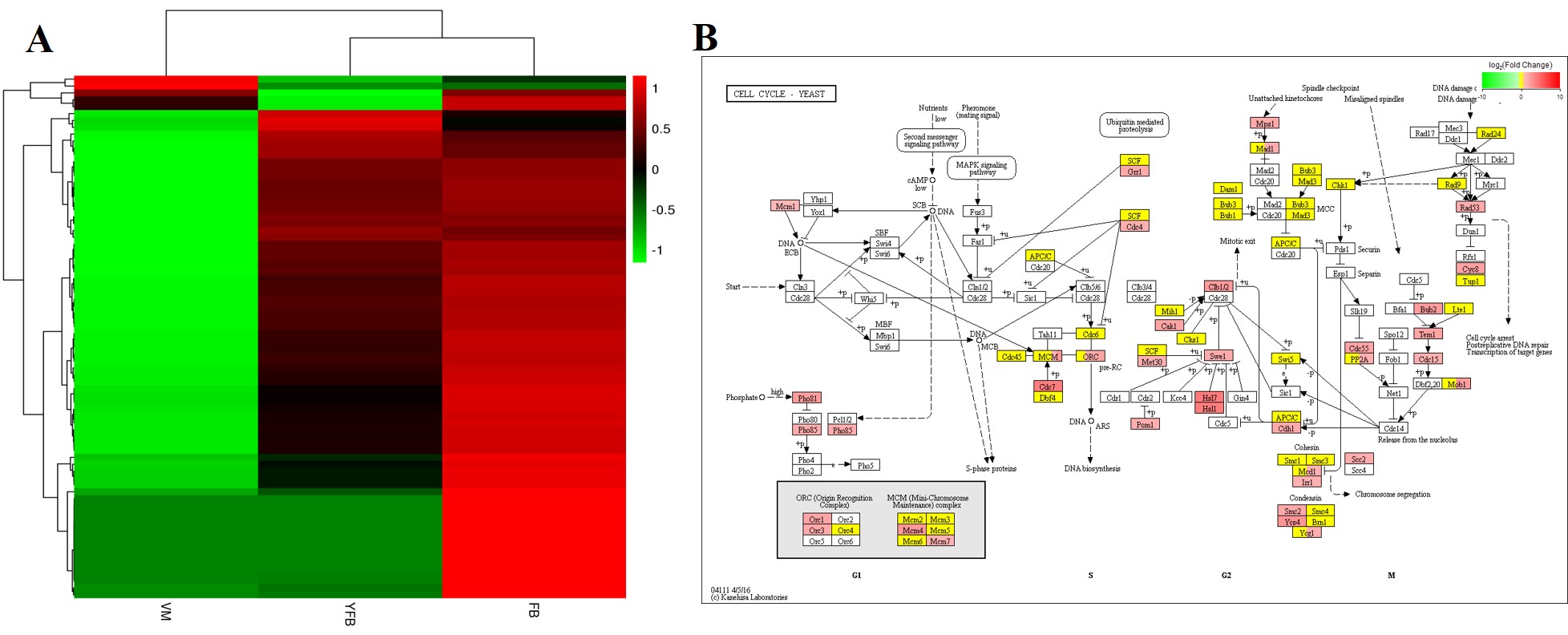


**Fig. S5 Heatmap analysis of unigenes involved in the cell-cycle and KEGG annotation of DEGs involved in the cell-cycle pathway.** (A)The heatmap shows the unigenes involved in the cell-cycle according to the KEGG database at the three stages. The color bar reflects gene expression levels, from green(low) to red (high). (B)DEGs between the VM and FB stages in the cell cycle pathway. Boxes with red frames represent up-regulated genes; boxes with yellow frames represent non-DEGs.
